# Structural and functional differences of gut microbiota in *Pomacea canaliculata* from different geographical locations and habitats

**DOI:** 10.1002/ece3.70283

**Published:** 2024-10-03

**Authors:** Miao Fang, Fandong Yu, Lu Shu, Hui Wei, Xidong Mu, Xuejie Wang, Meng Xu, Dangen Gu

**Affiliations:** ^1^ Pearl River Fisheries Research Institute Chinese Academy of Fishery Sciences Guangzhou China; ^2^ Key Laboratory of Prevention and Control for Aquatic Invasive Alien Species Ministry of Agriculture and Rural Affairs Guangzhou China; ^3^ Key Laboratory of Alien Species and Ecological Security (CAFS) Chinese Academy of Fishery Sciences Guangzhou China

**Keywords:** community characteristics, geospatial pattern, gut microorganism, habitats, *Pomacea canaliculata*

## Abstract

Gut microbiota is related to host fitness, and influenced by geographical locations and habitats. *Pomacea canaliculata* is a malignant invasive alien snail that threatens agricultural production and ecosystem functions worldwide. Clarifying the general rules of the gut microbial community structure and function of the snails in different geographical locations and habitats is of great significance for understanding their invasion at different spatial scales. This study used high‐throughput sequencing technology to compare and analyze the differences in community structure and function of gut microbiota in *P. canaliculata* from five geographical locations (Liuzhou, Yulin, Nanning, Wuzhou, and Hezhou) and three different habitats (pond, paddy field, and ditch) in Guangxi Province. The results showed that intestinal microbial alpha diversity of *P. canaliculata* was higher in Liuzhou, Yulin, lower in Nanning, Wuzhou, Hezhou, and higher in ponds compared with paddy fields and ditches. The dominant phyla of gut microbiota in snails were Firmicutes, Cyanobacteria, Proteobacteria, Fusobacteriota, Bacteroidota, and the dominant genus was *Lactococcus*. The community structure of gut microbiota in snails varied significantly across different geographical locations and habitats, and the phyla Firmicutes, Cyanobacteria had significantly higher relative abundance in snails collected from Nanning and Yulin, respectively. Moreover, the relative abundance of gut functional microbiota associated with human disease in *P. canaliculata* was significantly affected by geographical locations and habitats, and with the highest abundance in ponds. However, the relative abundance of functional microbiota related to metabolism, genetic information processing, organizational system, environmental information processing, and cellular processes were only significantly affected by geographical locations. Collectively, geographical locations and habitats had significantly different effects on the community structure and function of gut microbiota in *P. canaliculata*, and the greater differences were caused by geographical locations rather than by habitats.

## Graphical abstract

(1) Geographical locations and habitats had a significant effect on the community characteristics of gut microbiota in *Pomacea canaliculata*; (2) The greater effect was caused by geographical locations rather than by habitats.

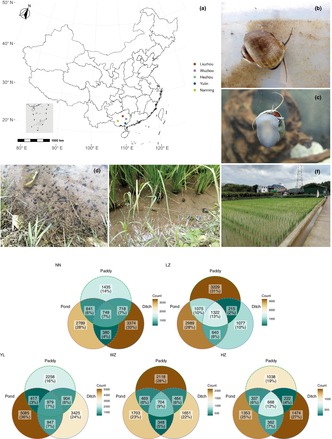

## 1 INTRODUCTION

*Pomacea canaliculata* is one of the 100 malignant invasive alien species in the world, which is native to the Amazon River Basin of South America. In Asia, *P. canaliculata*, as an important economic species of freshwater aquaculture, was once widely introduced. Later it is found that the damage of *P. canaliculata* to aquatic crops such as rice even exceeds the original plant diseases and insect pests, becoming one of the most serious pests for local crops (Halwart, 1994).

*Pomacea canaliculata* can be globally distributed, mainly because of its wide adaptability, strong ecological tolerance (Giraud‐Billoud et al., 2013), high reproduction rate (Tamburi & Martín, 2011), strong defense against natural enemies (Yoshie & Yusa, 2008), and other comprehensive reasons, which may enable them to obtain sufficient competitive advantages in new geographical locations and habitats. Different geographical locations and habitats always mean different food resources and microenvironments. *P. canaliculata* can well adapt to diverse geographical locations and habitats, indicating that their omnivory and ability to digest and absorb food play a vital role in this process, which may be closely associated with the structure and function of gut microbiota in themself. However, the geographical locations and habitats variation in structure and function of the gut microbiota in *P. canaliculata* has not yet been fully understood.

Gut microbiotas play a critical role in host immunity, growth, and disease resistance (Zheng et al., 2020). Previous studies are mainly focused on mammals (de Jonge et al., 2022), birds (Grond et al., 2018), fishes (Kim et al., 2021), and other animals with important economic value to human beings, while less attention is paid to invasive aquatic animals such as *P. canaliculata*. It has been known that the invasion range of exotic aquatic animals is mainly determined by climate factors, spatial heterogeneity, and habitats (Havel et al., 2015). Although there is little research on the role of geographical locations and habitats in modifying the gut microbiota of *P. canaliculata*, the difference of gut microbiota of *P. canaliculata* in different geographical locations and habitats may be mainly caused by various environments, which can be got inspirations from studies of other invasive aquatic animals. For example, compared with native frogs (*Lithobates clamitans*), the gut microbiota of American bullfrog (*Lithobates catesbeianus*) has stronger temperature plasticity (Fontaine & Kohl, 2020), thus increasing its ability to adapt to different environment. Another study suggests that gut microbiota of *Pacifastacus leniusculus* changes with the invasion range, and there is a significant difference in the gut microbiota of *P*. *leniusculus* between the invasion core area and the invasion front area (Dragicevic et al., 2021), indicating that changes in geographical locations have a significant effect on shaping its gut microbiota. A more direct example shows that after the invasion of *Potamopyrgus antipodarum* from New Zealand into Europe, although some core microbial groups are still preserved in the intestines, the gut microbiota of *P*. *antipodarum* undergoes significant changes, thus forming new gut microbiota symbiotes at the invasion site (Bankers et al., 2021). In addition, different habitats can also lead to differences in gut microbiota of invasive aquatic animals. For instance, there are significant differences in intestinal microbial diversity of *Oreochromis mossambicus* between rivers and lakes, suggesting that they may be able to obtain different bacteria from different environments they surrounded by (Gaikwad et al., 2017). The similar result is also found in the study of intestinal microbial diversity of *Procambarus clarkii* in reservoirs, paddy fields and rivers (Xavier et al., 2021). A more robust study suggests that the results of shotgun metagenomic sequencing of the digestive tract extract from *Achatina fulica* reveal multiple microbial genes through functional analysis. These genes can help the host degrade refractory lignocellulose, detoxify exogenous substances, synthesize essential amino acids, and vitamins, which may help it to adapt to different environments and maintain a wide range of food intake abilities (Cardoso et al., 2012). In summary, it can be viewed that the gut microbiota of invasive aquatic animals is mainly significantly affected by temperature, invasion range, geographical locations and habitats, or other factors, and they may change their gut microbiota to better adapt to the environment. So how about the change rule of gut microbiota in *P. canaliculata* during their invasion process?

Study has found that bacteria connected with digestive cellulose are enriched in the intestines of *P. canaliculata* from river (Li et al., 2019). The dominant phyla bacteria in the gut of *P. canaliculata* in paddy fields are Proteobacteria and Tenericute, the most abundant genera bacteria are *Aeromonas*, *Enterobacter*, *Desulfovibrio*, and *Citrobacter*, and among these genera, *Desulfovibrio* and *Citrobacter* bacteria have a special role in detoxifying heavy metals. The gut microbiota of *P. canaliculata* from ponds, including Bacteroidota, Actinobacteriota, Cyanobacteria, and Fusobacteria, will alternate and accumulate with the seasons to adapt to environmental changes (Li et al., 2022). Collectively, *P. canaliculata* can better adapt to diverse environments by altering its gut microbiota. However, there are some shortcomings in the researches of geographical locations and habitats effect on gut microbiota in *P. canaliculata*. Firstly, the sampling size and scale are insufficient, which range from a dozen to dozens and limit to a certain urban area. Secondly, the habitat for collecting samples is relatively single, most of the samples come from a single habitat thus lacking comparability. Thirdly, due to differences in sampling time and location between different studies, the results of researches about gut microbiota of *P. canaliculata* cannot be obtained uniformly understood. To make up for the shortcomings of the above researches, Guangxi Province, where the invasion of *P. canaliculata* is more severe, was selected for this study. Nested sampling method was adopted, and five geographical locations were selected within the province. Three sampling sites were randomly selected from each geographical location, with each sampling site containing three habitats. Five snails were collected from each habitat with five quadrats (one snail per quadrat), and a total of 225 adult snails were collected, and collecting intestines, exploring the effects of geographical locations and habitats on the structure and function of gut microbiota in *P. canaliculata* through high‐throughput sequencing of the 16S rRNA gene.

## 2 MATERIALS AND METHODS

### 2.1 Sample collection

**FIGURE 1:**
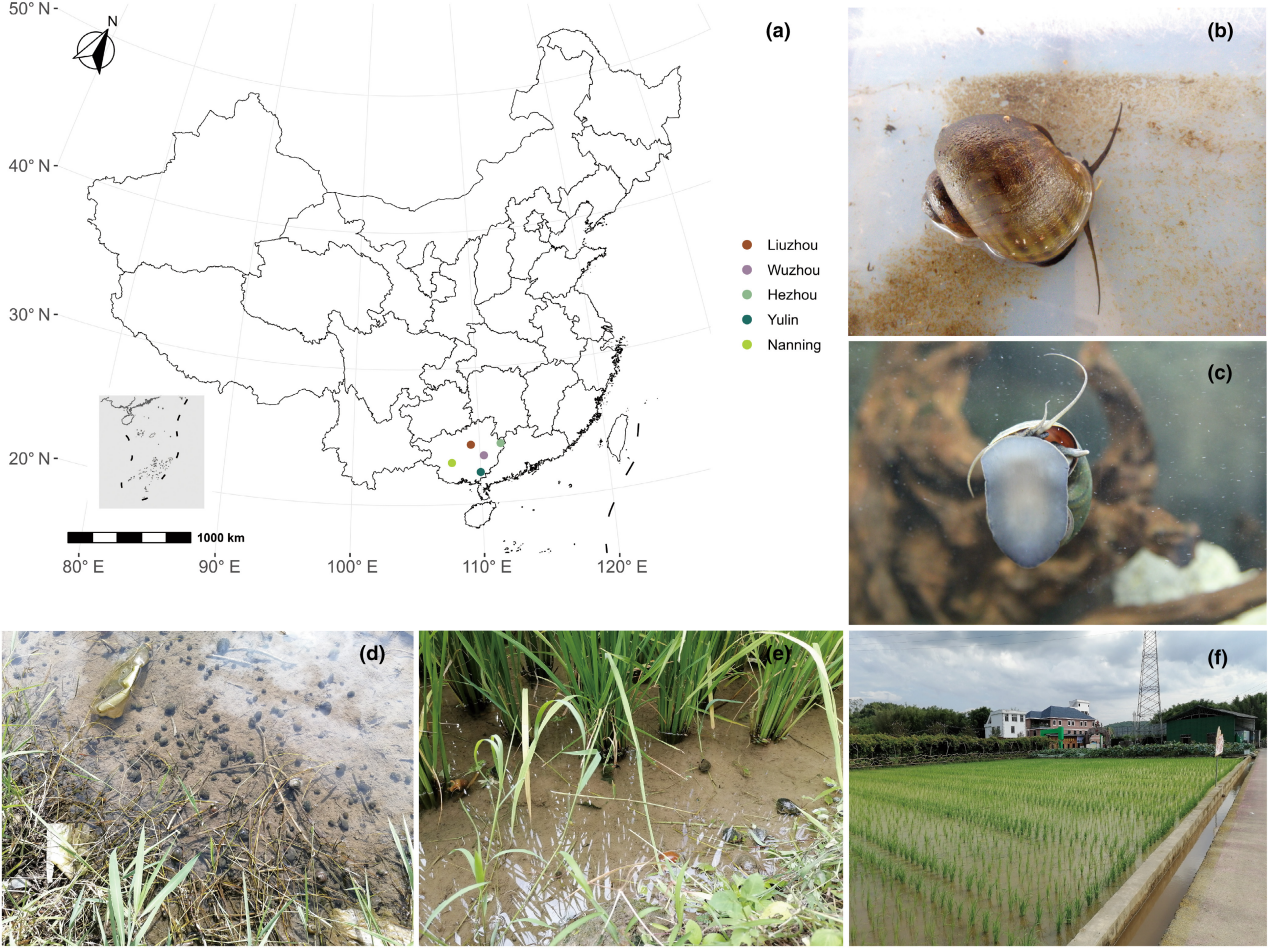
Sampling geographical location map and photos. (a) Sampling map presenting the geographical locations of five sampling points; (b) and (c) displaying the back and front photos of *Pomacea canaliculata*; (d–f) showing the pond, paddy field, and ditch photos of field sampling.

In August to September 2022, we collected *P. canaliculata* in the field from five geographical locations (Nanning, NN (107°77′ E, 23°09′ N); Liuzhou, LZ (109°31′ E, 24°37′ N); Yulin, YL (109°99′ E, 22°32′ N); Wuzhou, WZ (110°30′ E, 23°54′ N); Hezhou, HZ (111°67′ E, 24°35′ N)) in Guangxi Province, China (Figure 1a). Three sites contained three habitats (pond, paddy field, ditch, Figure 1d–f) simultaneously were randomly selected in each geographical location, and five quadrats (1 m^2^) were set in each habitat (Figure S3). One adult snail was taken from each quadrat for intestinal sample collection, and the distance between the sample quadrat was about 10 m. We distinguished between male and female when sampling the snails and 23 female snails and 22 male snails were collected from each geographical location. A total of 225 *P. canaliculata* were collected in this study (five geographical locations × three sites × three habitats × five replicates). All the testing snails collected from five geographical locations were preliminarily discerned by shell morphological analysis (Hayes et al., 2012) and using primers LCO1490 or HCO2198 to amplify cytochrome coxidase subunit I (COI) gene to identify *P. canaliculata* (Yang et al., 2019) which could be used to sequence for gut microbiota. The body weight, shell height (Table S2), shell width, and shell mouth width of each *P. canaliculata* were also measured. All sampling individuals were wiped with 75% ethanol three times and followed by rinsing twice in distilled water to sanitize the surface prior to dissection. The entire intestinal contents were extracted carefully to avoid rupturing the gut wall. Each sample was stored in a sterile tube using liquid nitrogen and later stored in a freezer of −80°C.

### 2.2 DNA extraction and sequencing

The total genomic DNA (gDNA) of each sample was extracted using the cetyltrimethylammonium bromide (CTAB) method (Allen et al., 2006). The V3–V4 hypervariable region of the 16S rDNA genes was amplified using specific bacterial primers 341F (CCTAYGGGRBGCASCAG) and 806R (GGACTACNNGGGTATCTAAT) by polymerase chain reactions (PCRs). All PCR mixtures contained 15 μL of Phusion® High‐Fidelity PCR Master Mix (New England Biolabs), 0.2 μM of each primer and 10 ng target DNA, and cycling conditions consisted of a first denaturation step at 98°C for 1 min, followed by 30 cycles of denaturation at 98°C for 10 s, primer annealing at 50°C for 30 s, and extension at 72°C for 30 s, with a final extension step carried out at 72°C for 5 min to ensure complete amplification. The PCR products were purified with a Qiagen Gel Extraction Kit (Qiagen, Germany). Sequencing libraries were generated with NEBNext® Ultra™ IIDNA Library Prep Kit (Cat No. E7645) following manufacturer's recommendations, and the library quality was evaluated on the Qubit@ 2.0 Fluorometer (Thermo Scientific) and Agilent Bioanalyzer 2100 system. The library was sequenced on an Illumina NovaSeq platform.

### 2.3 Statistical and bioinformatics analyses

Firstly, paired‐end reads were assigned to samples based on their unique barcodes and were truncated by cutting off the barcodes and primer, and merged using FLASH (Version 1.2.11). Quality filtering on the raw tags were performed using the fastp (Version 0.20.0) software to obtain high‐quality clean tags which were compared with the SILVA 123 database using Vsearch (Version 2.15.0) to detect the chimera sequences, and the chimera sequences were removed to obtain the effective tags. Denoise of the effective tags was performed with DADA2 to obtain initial amplicon sequence variants (ASVs), and then ASVs with abundance less than 5 were filtered out. Secondly, species annotation was performed using QIIME2 software (Version QIIME2‐202006) based on SILVA 123 database and multiple sequence alignment was performed to study phylogenetic relationship of each ASV and the differences of the dominant species among different samples. Finally, all samples were rarefied to the sequencing depth of the lowest sample (26,970 clean reads).

Alpha diversity indices including the Shannon, Simpson, and Chao1 indices were calculated based on the output normalized data using “diversity” function in vegan package. Linear mixed‐effects models (LMMs) were used to analyze the differences in alpha diversity among geographical locations and habitats with geographical locations, habitats and their interaction as fixed effect and sites as a random effect. The models were generated in R with the lme4 and lmerTest packages and multiple comparisons were performed between each level of geographical locations and habitats using the “emmeans” function in the emmeans package, with Tukey's adjustment method for multiple comparisons at α = .05. Composition of gut microbiota in different geographical locations and habitats at phylum and genus level was displayed by stacked bar chart using ggplot2 package. We then analyzed the gut microbial community composition dissimilarities among the geographical locations and habitats by performing permutational multivariate analysis of variance (PERMANOVA) using the “adonis” function. The non‐metric multidimensional scaling (NMDS) method was used to analyze gut microbial community composition with the “metaMDS” function, and the Bray‐Curtis distance was calculated using the “vegdist” function. All the analyses were performed in the vegan package. A Venn diagram was generated to describe unique and common ASVs among different habitats under each geographical location using ggVennDiagram package. The LEfSe software (Version 1.0) was used to do linear discriminant analysis effect size (LEfSe) analysis (|LDA score| > 4) to find out the significant biomarkers among geographical locations and habitats (*p* < .05). Further, in order to study the functions of the communities in the samples and find out the different functions of the communities in the different geographical locations and habitats, based on the SILVA 123 database and the Kyoto Encyclopedia of Genes and Genomes (KEGG) database, the Tax4Fun package was used for predicting the abundance of functional microbiota associated with KEGG pathways in intestinal bacterial communities. Relative abundance bar chart of gut microbiota KEGG pathways at first and second level was presented, and the relative abundance differences at first level across different geographical locations and habitats were also analyzed using LMMs as similar as the difference for alpha diversity analysis.

## 3 RESULTS

### 3.1 Sequencing depth and alpha diversity indices

DNA extracted from 225 *P. canaliculata* samples was amplified successfully, and 13,983,287 effective tags were obtained. *P. canaliculata* yielded 34,217 valid ASVs which were assigned into 56 phyla, 148 classes, 358 orders, 615 families, and 1508 genera. The rarefaction curves for all the samples showed that sequences number plateaued at approximately 26,970 sequences (Figure S1), indicating this sequencing depth was sufficient for subsequent analyses.

**FIGURE 2:**
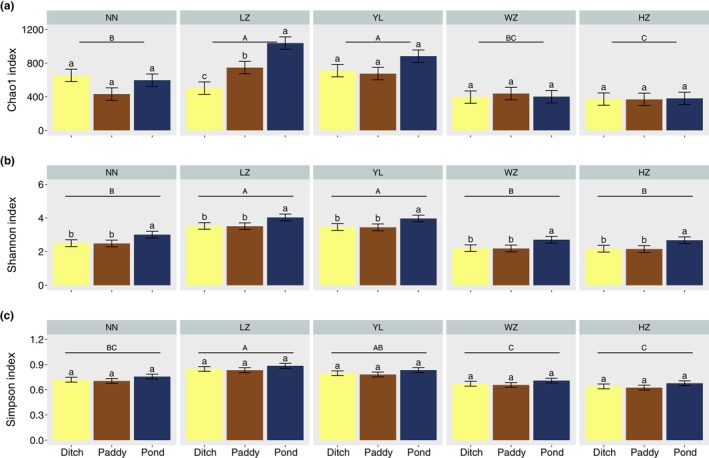
Comparison of alpha diversity index of gut microbiota in *Pomacea canaliculata* under different geographical locations and habitats. (a) Chao1 index; (b) Shannon index; (c) Simpson index. Differences between geographical locations are indicated by different capital letters (*p* < .05), and differences between habitats are marked as different lowercase letters (*p* < .05).

The gut microbial alpha diversity (Chao1, Shannon and Simpson index) was significantly influenced by geographical locations and habitats (*p* < .05) (Table S1). When considering the geographical locations and habitats separately, the trends of the Chao1, Shannon, and Simpson index were LZ > YL > NN > WZ > HZ and pond > paddy > ditch, respectively (Figure 2). In addition, Chao1 and Shannon index were also influenced by the interactions between geographical locations and habitats (Figure 2a,b) while not for Simpson index (Figure 2c).

### 3.2 Taxonomic composition and beta diversity analysis

**FIGURE 3:**
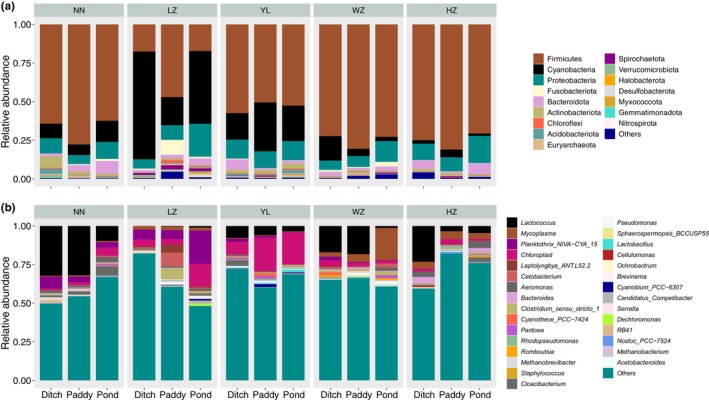
Relative abundance of gut microbiota in *Pomacea canaliculata* from different geographical locations and habitats. (a) Only mean phyla with relative abundance >0.01% was showed; (b) Relative abundance of top 30 genera was showed.

The dominant gut microbiota of *P. canaliculata* at the phylum level showed the similar trend in different geographical locations and habitats, but the relative proportion of each microbiota was different (Figure 3a). The dominant bacterial phyla detected within gut microbiota of *P. canaliculata* in the five geographical locations and three habitats were Firmicutes (varied from 17.2% to 81.1%), Cyanobacteria (1.5% to 69.8%), Proteobacteria (5.9% to 21.3%), Fusobacteriota (0.06% to 9.6%), and Bacteroidota (0.9% to 8.3%). Except for these five phyla, another 11 phyla were also presented, including Actinobacteriota, Chloroflexi, Acidobacteriota, Euryarchaeota, Spirochaetota, Verrucomicrobiota, Halobacterota, Desulfobacterota, Myxococcota, Gemmatimonadota, and Nitrospirota. The intestinal microbiota of *P. canaliculata* at the genus level varied greatly in different geographical locations and habitats (Figure 3b). Specifically, the relative abundance of the top 30 genera in the gut ranged from 17.81% to 51.85%, with *Lactococcus* species being dominant in different habitats in NN (24.62%) and HZ (10.43%), as well as in paddy fields and ditches in WZ (17.68%). The top two abundance genera in the intestines of *P. canaliculata* in LZ were *Planktothrix_NIVA‐CYA_15* and *Chloroplast*, while those in YL were *Chloroplast* and *Lactococcus*. Additionally, all other genera were presented in relative abundances <1% across all samples.

**FIGURE 4:**
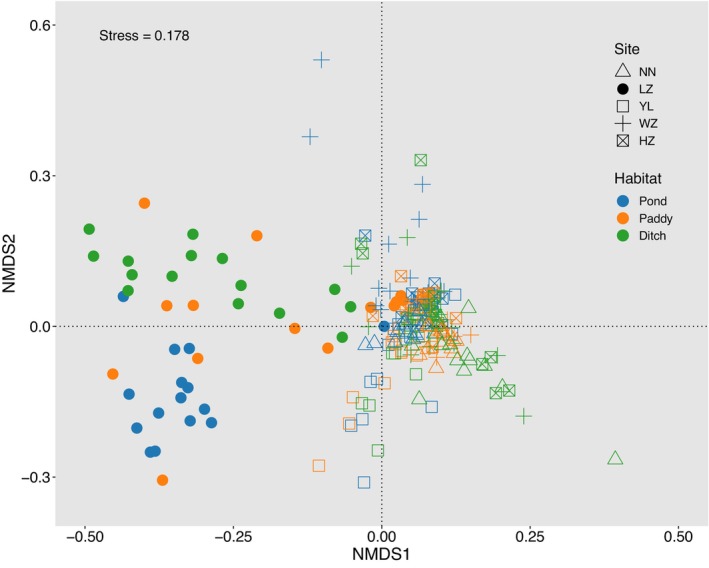
Non‐metric multidimensional scaling (NMDS) analysis based on Bray‐Curtis distances of gut microbiota in *Pomacea canaliculata* from different geographical locations and habitats. Permutational multivariate analysis of variance (PERMANOVA) shows significant dissimilarities of intestinal microbial community structures of *P. canaliculata* from different geographical locations and habitats (*p* < .05; permutation time = 999).

The community compositions of gut microbiota in *P. canaliculata* were significantly, only mildly influenced by geographical locations (*F* = 21.26, *R* ^2^ = .24, *p* = .001), habitats (*F* = 8.09, *R* ^2^ = .05, *p* = .001), and their interactions (*F* = 5.46, *R* ^2^ = .12, *p* = .001), which might result in spatial and habitat differences (Figure 4).

### 3.3 Different gut microbiota in *P. canaliculata* from different geographical locations and habitats

**FIGURE 5:**
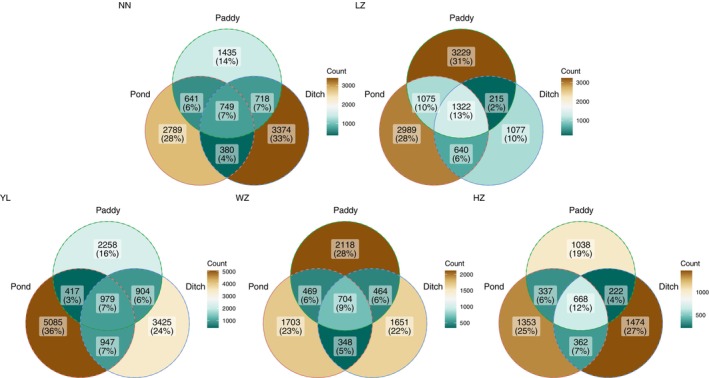
Venn diagram shows the numbers of common and unique ASVs among the pond, paddy, and ditch under five geographical locations.

Gut microbiota in *P. canaliculata* from pond, paddy, and ditch shared between 668 and 1322 ASVs in five geographical locations. There were also unique ASVs with a range from 1353 to 5085 in pond, 1038 to 3229 in paddy, and 1077 to 3425 in ditch under five geographical locations (Figure 5).

**FIGURE 6:**
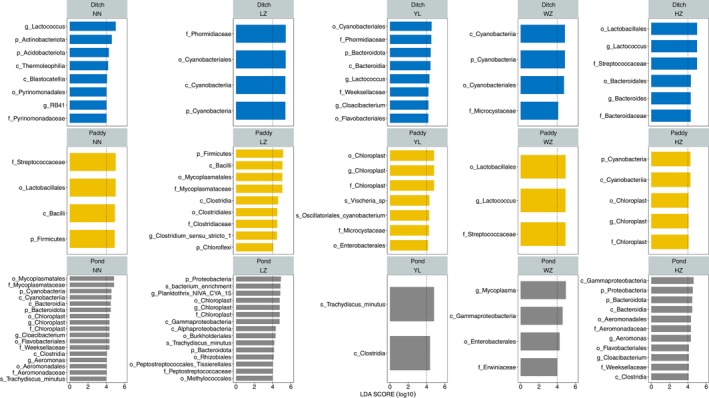
Comparisons of gut microbiota variations of *Pomacea canaliculata* from different habitats in five geographical locations using LEfSe analysis, respectively. The LDA value bar chart shows the species with LDA scores greater than the set value (|LDA| > 4), which are biomarkers with statistical differences between habitats (*p* < .05). The length of the bar chart represents the effect of different species (i.e., LDA score). The p, c, o, f, g, s in diagram represents phylum, class, order, family, genus, and species, respectively.

Linear discriminant analysis effect size (LEfSe) analysis was performed to identify biomarkers that differed significantly among three habitats under five geographical locations. From the bar chart of LDA effect value, the intestinal microbiota of *P. canaliculata* from NN showed that Actinobacteriota, *Lactococcus* were enriched in ditch; Firmicutes, Bacilli, Lactobacillales, and Streptococcaceae were enriched in paddy; Cyanobacteria, Cyanobacteriia, Mycoplasmatales, Mycoplasmataceae, *Cloacibacterium*, were enriched in pond. *P. canaliculata* from LZ revealed that Cyanobacteria, Cyanobacteriia, Cyanobacteriales, and Phormidiaceae were enriched in ditch; Firmicutes, Bacilli, Mycoplasmatales, Mycoplasmataceae, and *Clostridium_sensu_stricto_1* were enriched in paddy; Proteobacteria, Gammaproteobacteria, Chloroplast, Chloroplast, *Planktothrix_NIVA_CYA_15*, and *bacterium_enrichment* were enriched in pond. *P. canaliculata* from YL indicated that Bacteroidota, Bacteroidia, Phormidiaceae, and *Lactococcus* were enriched in ditch; Chloroplast, Chloroplast, *Chloroplast*, and *Vischeria_sp* were enriched in paddy; Clostridia, *Trachydiscus_minutus* were enriched in pond. *P. canaliculata* from WZ manifested that Cyanobacteria, Cyanobacteriia, Cyanobacteriales, and Microcystaceae were enriched in ditch; Lactobacillales, Streptococcaceae, and Lactococcus were enriched in paddy; Gammaproteobacteria, Enterobacterales, Erwiniaceae, and *Mycoplasma* were enriched in pond. *P. canaliculata* from HZ demonstrated that Lactobacillales, Streptococcaceae, and *Lactococcus* were enriched in ditch; Cyanobacteria, Cyanobacteriia were enriched in paddy; Proteobacteria, Gammaproteobacteria, Aeromonadales, Aeromonadaceae, and *Aeromonas* were enriched in pond (Figure 6).

### 3.4 Functional prediction of intestinal bacteria in *P. canaliculata*

**FIGURE 7:**
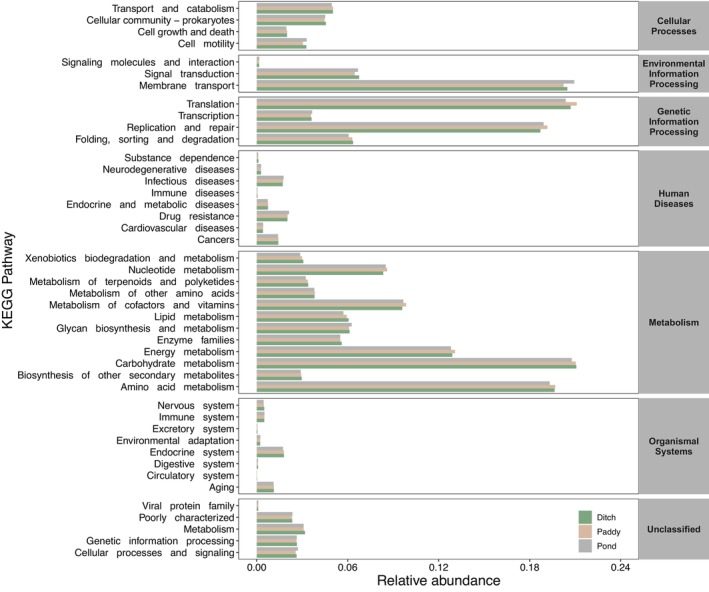
KEGG pathway annotation of gut microbiota in *Pomacea canaliculata* from different geographical locations and habitats. The bar chart represents the mean relative abundance of KEGG pathway annotation at level 2 across the different habitats.

**FIGURE 8:**
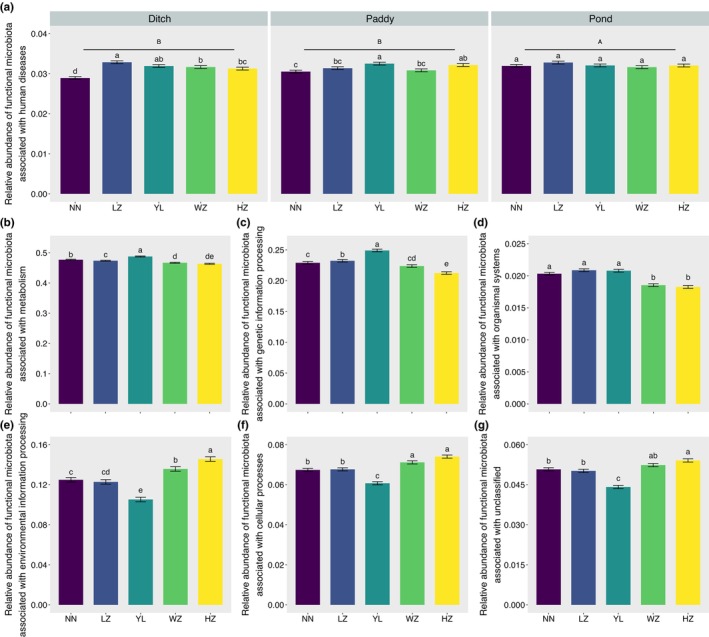
The effects of geographical locations and habitats on relative abundance of differentially functional gut microbiota associated with (a: Human diseases; b: Metabolism; c: Genetic information processing; d: Organismal systems; e: Environmental information processing; f: Cellular processes; g: Unclassified) in *Pomacea canaliculata*. The different uppercase letters in (a) or lowercase letters in (b, c, d, e, f, g) denote significant difference (*p* < .05).

A total of 6632 KEGG Orthology (KOs) were mapped to 390 level three KEGG pathways and were then classified into 44 level 2 KEGG pathways (Figure 7). Of these 44 secondary KEGG pathways, 12 pathways related to metabolism, eight pathways related to organismal systems, eight pathways related to human diseases, five pathways related to unclassified functions, four pathways related to cellular processes, four pathways related to genetic information processing, and three pathways related to environmental information processing. At level 1, metabolism accounted for the highest proportion of predicted functional pathways, followed by genetic information processing, environmental information processing, human diseases, cellular processes, unclassified, and organismal systems (Figure 7). At level 2, the abundance of functional microbiota related to replication and repair, amino acid metabolism, translation, membrane transport, carbohydrate metabolism at global and overview maps were relatively high (Figure 7). In order to further compare whether there were differences in the abundance of intestinal functional microbiota in *P. canaliculata* from different geographical locations and habitats, the results of LMMs showed that the abundance of functional microbiota associated with human diseases in the gut was significantly influenced by geographical locations and habitats (Figure 8a; Table S1), while the abundances of functional microbiota related to metabolism (Figure 8b), genetic information processing (Figure 8c), organismal systems (Figure 8d), environmental information processing (Figure 8e), cellular processes (Figure 8f), and unclassified (Figure 8g) were only significantly influenced by geographical locations (Table S1).

## 4 DISCUSSION

Alpha diversity of gut microbiota in *P. canaliculata* was significantly influenced by geographical locations and habitats. Except for the Chao1 index of gut microbiota from LZ, which was significantly influenced by habitats, the rest indexes were only significantly affected by geographical locations. The reasons probably were that Chao1 index mainly reflects the number of microbial species in the intestines of *P. canaliculata*, while the Shannon and Simpson index consider both the richness and evenness of gut microbial communities (Thukral, 2017). Moreover, the Shannon index puts more weight into richness and the Simpson index puts more weight into evenness. Additionally, alpha diversity of gut microbiota in *P. canaliculata* was relatively higher in ponds, and the role of geographical locations in modifying the species diversity of gut microbiota in *P. canaliculata* was consistent. On the one hand, it might because *P. canaliculata* often wintered in ponds and had a longer lifespan, resulting in the larger sampling body weight (Li et al., 2022; Qin et al., 2020; Tamburi & Martín, 2009), which might have a positive correlation with the alpha diversity. On the other hand, pond probably had the stable water environment, ultimately leading to a higher diversity of gut microbiota in *P. canaliculata* (Bankers et al., 2021; Dulski et al., 2020; Xavier et al., 2021). Compared to habitats, geographical locations had a greater effect on the gut microbiota of *P. canaliculata*, probably indicating that the gut microbiota of *P. canaliculata* was influenced by both external environment (Dragicevic et al., 2021; Dulski et al., 2020; Picazo et al., 2020) and the host itself (Kim et al., 2021; Xavier et al., 2021).

The community composition of gut microbiota in *P. canaliculata* varied greatly by geographical locations and habitats. The top five phyla were Firmicutes, Cyanobacteria, Proteobacteria, Bacteroidota, and Actinobacteriota, which was in line with the results of study about season effects on gut microbiota in *P. canaliculata* (Li et al., 2022). To our knowledge, many members of the Firmicutes were beneficial bacteria that played an important role in maintaining gut microbiota health and promoting homeostasis of hosts (Ben David et al., 2015); The Firmicutes was also closely related to the degradation process of cellulose (Sommer et al., 2016), which was beneficial for omnivorous snails to extensively digest herbivorous foods (Zhou et al., 2022). Moreover, the high‐frequency detection of cyanobacteria in the intestines might be due to feeding of large amounts of algae by *P. canaliculata* (Fang et al., 2010; Xie et al., 2021), while the high abundance of Proteobacteria microorganisms, because of their ability to secrete lipase, protease, and amylase, were directly connected with multiple metabolic pathways of the host (Nicolai et al., 2015; Pawar et al., 2015), thereby affecting the host's environmental adaptability. The Bacteroidota was closely correlated with the fermentation of sugars and the utilization of nitrogen‐containing substances (Jami et al., 2013; Zhou et al., 2022), while the Actinobacteriota microorganisms often existed in soil and animal bodies, playing a vital role in the decomposition of organic matter (Binda et al., 2018; Servin et al., 2007). The differences between the results of this study and other similar studies might mainly be caused by different sampling geographical locations (Dragicevic et al., 2021), seasons (Dulski et al., 2020), and habitats (Jing et al., 2021; Kang et al., 2022). Although the five phyla mentioned above accounted for more than 90% of the richness of gut microbiota in *P. canaliculata*, further verification was needed to determine whether the changes in gut microbiota across different geographical locations and habitats could reflect their adaptability to different environments (Liu et al., 2023). In addition, the analysis results of NMDS based on Bray‐Curtis distance showed that the community structure of gut microbiota in *P. canaliculata* was significantly influenced by geographical locations and habitats. Geographical locations explained about 24% of the variation in community structure, habitats explained about 5%, and the interaction between the two explained about 12%, The effect of geographical locations on the structure of gut microbiota was greater than that of habitats (Lilli et al., 2023; Wilson et al., 2019), indicating that climate factors (Picazo et al., 2020; Sepulveda & Moeller, 2020) or anthropogenic factors (Stock et al., 2021) might play a greater role in shaping the structure of gut microbiota in *P. canaliculata* than food composition (Ringø et al., 2015) or the host itself (Kim et al., 2021). For example, seasonal changes (mainly temperature changes) had a significant impact on the structure of gut microbiota in *P. canaliculata* (Li et al., 2022), thereby affecting their functions (Yuan et al., 2021). Different habitats (different food compositions and microenvironments) probably mainly affected the abundance of gut microbiota, and the impact on its community composition was not as significant as climate factors.

The alpha diversity, composition and beta diversity of gut microbiota in *P. canaliculata* varied greatly among different geographical locations and habitats. However, it was also found that there were 7%–13% common gut microbiota among different habitats within the same geographical location, indicating that there might be a core microbial community in the gut microbiota of *P. canaliculata*, and the gut microbiota also had a certain host dependence (Kokou et al., 2019; Rudi et al., 2018; Zhang et al., 2022). On the one hand, it might be due to the presence of certain connectivity between ponds, paddy fields, and ditches during the sampling process in the same geographical location, which weakened the sampling environmental heterogeneity. On the other hand, the circulation of *P. canaliculata* between different habitats might ultimately lead to the emergence of shared gut microbiota. In addition, there were some unique gut microorganisms in different geographical locations and habitats. In the same habitat, *P. canaliculata* from different geographical locations had enriched gut microbiota of different phyla (Actinobacteriota, Firmicutes, Cyanobacteria, Bacteroidota, Chloroflexi, Proteobacteria, one phylum, or several phyla of them) while in the same geographical locations, the microbial phyla enriched in the intestines of *P. canaliculata* from different habitats had similarities and differences, and the enriched gut microbiota were mainly related to the food types they fed on, as well as digestion, absorption, and metabolism. These results suggested that *P. canaliculata* changed its gut microbiota to better adapt to the environment (Bae et al., 2021; Li et al., 2022). It was worth mentioning that gut microbiota of *P. canaliculata* from ponds had relatively more enriched phyla in a single geographical location, and there were large differences among different geographical locations. Combined with the higher alpha diversity in gut microbiota of *P. canaliculata* from ponds, the reason might be owing to the size of the sampled *P. canaliculata* (Chen et al., 2021). Linear regression analysis between Shannon index and the body weight, shell height of *P. canaliculata* indeed confirmed a weak and positive correlation between them. However, in some geographical locations, this positive correlation had not reached a significant level (Figure S2), indicating that this enrichment effect was also caused by other factors, such as host identity (Hu et al., 2024) and water microenvironment (Kirchoff et al., 2022). Furthermore, ponds are one of the important wintering sites for *P. canaliculata* in southern China. Exploring the enrichment patterns and driving factors of gut microbiota in this habitat is of great guiding significance for exploring the invasion mechanism and prevention and control of *P. canaliculata*, and this is also a research topic that needs to be focused on in the future study.

Different geographical locations and habitats also had significant impacts on the function of gut microbiota in *P. canaliculata*. In this study, it was found that the relative abundance of functional microbiota related to carbohydrate metabolism, amino acid metabolism, energy metabolism, transcription, replication and repair, membrane transport, and other metabolic pathways in the intestines of *P. canaliculata* were enriched. The analysis results of LMMs found that KEGG pathways at level 1, relative abundance of predicted functional microbiota associated with human disease in gut of *P. canaliculata* were significantly influenced by geographical locations and habitats. Overall, the relative abundance of these gut microbiota was higher in *P. canaliculata* from ponds. Although the underlying reasons needed further exploration, it was concerning whether they might exist a risk of some infectious diseases. At the same time, the connectivity of habitats was the main reason for the difficulty in controlling *P. canaliculata*, and the relatively stable water environment in ponds was one of the main wintering sites for *P. canaliculata*. Hence, strengthening the supervision of ponds and other wintering sites for *P. canaliculata* might be an important direction for their prevention and control (Carlsson et al., 2004; Fang et al., 2010; Liu et al., 2020). In addition, the relative abundance of predicted functional microbiota associated with metabolism, genetic information processing, and organizational system showed a significant downward trend among YL, WZ, and HZ, respectively, while the relative abundance of predicted functional microbiota associated with environmental information processing and cellular process showed the opposite trend. Further network analysis was needed to verify the potential antagonistic effect between some gut microbiota of *P. canaliculata*. It should be noted that this study aimed to describe the general pattern of gut microbiota structure and function in *P. canaliculata*, and did not explore the potential influencing factors that formed the patterns. Subsequent researches would focus on clarifying the key factors that affected the characteristics of the gut microbiota community in *P. canaliculata*, determining whether there were general patterns in the assembly of gut microbiota community in *P. canaliculata* at different spatial scales, determining the driving factors, and examining the effects of the community assembly of gut microbiota on their invasion. Based on these analyses to establish the link between the characteristics of the gut microbiota community of *P. canaliculata* and their invasion.

In summary, significant differences were found in the structure and function of the gut microbiota of *P. canaliculata* across different geographical locations and habitats. The effects of geographical locations on gut microbiota of *P. canaliculata* were stronger than those of habitats. The results indicated that variations in climate factors caused by geographical locations change had more important impacts than those of variations in environmental factors caused by habitats change on the gut microbiota of *P. canaliculata*. This study will provide a general understanding the adaptive strategies of gut microbiota in *P. canaliculata* to geographical locations and habitats change. Further study will focus on the interaction between the gut microbiota and the host, co‐occurrence network relationship and the assembly of intestinal microbial community in *P. canaliculata* at different spatial scales.

## AUTHOR CONTRIBUTIONS

**Miao Fang:** Formal analysis (equal); investigation (equal); methodology (equal); writing – original draft (equal). **Fandong Yu:** Formal analysis (equal); investigation (equal); resources (equal); writing – review and editing (equal). **Lu Shu:** Methodology (equal); writing – review and editing (equal). **Hui Wei:** Investigation (equal); writing – review and editing (equal). **Xidong Mu:** Project administration (equal); supervision (equal). **Xuejie Wang:** Data curation (equal); software (equal). **Meng Xu:** Funding acquisition (equal); methodology (equal); visualization (equal). **Dangen Gu:** Conceptualization (lead); supervision (lead).

## FUNDING INFORMATION

This study was supported by fundings from the China Agriculture Research System of MOF and MARA (No. CARS‐45), the Science and Technology Program of Guangzhou, China (No. 202201010257; 2023B03J1306), the Science and Technology Program of Guangdong, China (No. 2023A1414020001), the Central Public‐interest Scientific Institution Basal Research Fund, CAFS (No. 2023TD17; 2024SJRC12).

## CONFLICT OF INTEREST STATEMENT

The authors declare no competing interests.

## Supporting information


Data S1.

## Data Availability

The experiment datasets in the article can be accessed at Dryad Digital Repository: https://doi.org/10.5061/dryad.4f4qrfjmk.

## References

[ece370283-bib-0001] Allen, G. C. , Flores‐Vergara, M. A. , Krasnyanski, S. , Kumar, S. , & Thompson, W. F. (2006). A modified protocol for rapid DNA isolation from plant tissues using cetyltrimethylammonium bromide. Nature Protocols, 1(5), 2320–2325. 10.1038/nprot.2006.384 17406474

[ece370283-bib-0002] Bae, M. J. , Kim, E. J. , & Park, Y. S. (2021). Comparison of invasive apple snail (*Pomacea canaliculata*) behaviors in different water temperature gradients. Water, 13(9), 1149. 10.3390/w13091149

[ece370283-bib-0003] Bankers, L. , Dahan, D. , Neiman, M. , Adrian‐Tucci, C. , Frost, C. , Hurst, G. D. D. , & King, K. C. (2021). Invasive freshwater snails form novel microbial relationships. Evolutionary Applications, 14(3), 770–780. 10.1111/eva.131-58 33767751 PMC7980272

[ece370283-bib-0004] Ben David, Y. , Dassa, B. , Borovok, I. , Lamed, R. , Koropatkin, N. M. , Martens, E. C. , White, B. A. , Bernalier‐Donadille, A. , Duncan, S. H. , Flint, H. J. , Bayer, E. A. , & Moraïs, S. (2015). Ruminococcal cellulosome systems from rumen to human. Environmental Microbiology, 17(9), 3407–3426. 10.1111/1462-2920.12868 25845888

[ece370283-bib-0005] Binda, C. , Lopetuso, L. R. , Rizzatti, G. , Gibiino, G. , Cennamo, V. , & Gasbarrini, A. (2018). Actinobacteria: A relevant minority for the maintenance of gut homeostasis. Digestive and Liver Disease, 50(5), 421–428. 10.1016/j.dld.2018.02.012 29567414

[ece370283-bib-0006] Cardoso, A. M. , Cavalcante, J. J. V. , Cantao, M. E. , Thompson, C. E. , Flatschart, R. B. , Glogauer, A. , Scapin, S. M. N. , Sade, Y. B. , Beltrao, P. J. M. S. I. , Gerber, A. L. , Martins, O. B. , Garcia, E. S. , de Souza, W. , & Vasconcelos, A. T. R. (2012). Metagenomic analysis of the microbiota from the crop of an invasive snail reveals a rich reservoir of novel genes. PLoS One, 7(11), e48505. 10.1371/journal.pone.0048505 23133637 PMC3486852

[ece370283-bib-0007] Carlsson, N. O. L. , Bronmark, C. , & Hansson, L. A. (2004). Invading herbivory: The golden apple snail alters ecosystem functioning in Asian wetlands. Ecology, 85(6), 1575–1580. 10.1890/03-3146

[ece370283-bib-0008] Chen, L. , Li, S. X. , Xiao, Q. , Lin, Y. , Li, X. X. , Qu, Y. F. , Wu, G. G. , & Li, H. (2021). Composition and diversity of gut microbiota in *Pomacea canaliculata* in sexes and between developmental stages. BMC Microbiology, 21(1), 200. 10.1186/s12866-021-02259-2 34210255 PMC8252327

[ece370283-bib-0009] de Jonge, N. , Carlsen, B. , Christensen, M. H. , Pertoldi, C. , & Nielsen, J. L. (2022). The gut microbiome of 54 mammalian species. Frontiers in Microbiology, 13, 886252. 10.3389/fmicb.2022.886252 35783446 PMC9246093

[ece370283-bib-0010] Dragicevic, P. , Bielen, A. , Petric, I. , Vuk, M. , Zucko, J. , & Hudina, S. (2021). Microbiome of the successful freshwater invader, the signal crayfish, and its changes along the invasion range. Microbiology Spectrum, 9(2), e0038921. 10.1128/Spectrum.00389-21 34494878 PMC8557874

[ece370283-bib-0011] Dulski, T. , Kozlowski, K. , & Ciesielski, S. (2020). Habitat and seasonality shape the structure of tench (*Tinca tinca* L.) gut microbiome. Scientific Reports, 10(1), 4460. 10.1038/s41598-020-61351-1 32157130 PMC7064478

[ece370283-bib-0012] Fang, L. , Wong, P. K. , Lin, L. I. , Lan, C. , & Qiu, J.‐W. (2010). Impact of invasive apple snails in Hong Kong on wetland macrophytes, nutrients, phytoplankton and filamentous algae. Freshwater Biology, 55(6), 1191–1204. 10.1111/j.1365-2427.2009.02343.x

[ece370283-bib-0013] Fontaine, S. S. , & Kohl, K. D. (2020). Gut microbiota of invasive bullfrog tadpoles responds more rapidly to temperature than a noninvasive congener. Molecular Ecology, 29(13), 2449–2462. 10.1111/mec.15487 32463954

[ece370283-bib-0014] Gaikwad, S. S. , Shouche, Y. S. , & Gade, W. N. (2017). Deep sequencing reveals highly variable gut microbial composition of invasive fish Mossambicus tilapia (*Oreochromis mossambicus*) collected from two different habitats. Indian Journal of Microbiology, 57(2), 235–240. 10.1007/s12088-017-0641-9 28611502 PMC5446831

[ece370283-bib-0015] Giraud‐Billoud, M. , Vega, I. A. , Tosi, M. E. R. , Abud, M. A. , Calderón, M. L. , & Castro‐Vazquez, A. (2013). Antioxidant and molecular chaperone defences during estivation and arousal in the south American apple snail. Journal of Experimental Biology, 216(4), 614–622. 10.1242/jeb.075655 23077161

[ece370283-bib-0016] Grond, K. , Sandercock, B. K. , Jumpponen, A. , & Zeglin, L. H. (2018). The avian gut microbiota: Community, physiology and function in wild birds. Journal of Avian Biology, 49(11), e01788. 10.1111/jav.01788

[ece370283-bib-0017] Halwart, M. (1994). The golden apple snail *Pomacea canaliculatain* Asian rice farming systems: Present impact and future threat. International Journal of Pest Management, 40(2), 199–206. 10.1080/09670879409371882

[ece370283-bib-0018] Havel, J. E. , Kovalenko, K. E. , Thomaz, S. M. , Amalfitano, S. , & Kats, L. B. (2015). Aquatic invasive species: Challenges for the future. Hydrobiologia, 750(1), 147–170. 10.1007/s10750-014-2166-0 32214452 PMC7087615

[ece370283-bib-0019] Hayes, K. A. , Cowie, R. H. , Thiengo, S. C. , & Strong, E. E. (2012). Comparing apples with apples: Clarifying the identities of two highly invasive Neotropical Ampullariidae (Caenogastropoda). Zoological Journal of the Linnean Society, 166(4), 723–753. 10.1111/j.1096-3642.2012.00867.x

[ece370283-bib-0020] Hu, Z. , Tong, Q. , Chang, J. , Xu, J. , Wu, B. , Han, Y. , Yu, J. , & Niu, H. (2024). Host species of freshwater snails within the same freshwater ecosystem shapes the intestinal microbiome. Frontiers in Ecology and Evolution, 12, 1359. 10.3389/fevo.2024.1341359

[ece370283-bib-0021] Jami, E. , Israel, A. , Kotser, A. , & Mizrahi, I. (2013). Exploring the bovine rumen bacterial community from birth to adulthood. ISME Journal, 7(6), 1069–1079. 10.1038/ismej.2013.2 23426008 PMC3660679

[ece370283-bib-0022] Jing, X. , Su, S. , Zhang, C. , Zhu, J. , Hou, Y. , Li, Z. , Yang, X. L. , Zhou, X. L. , He, X. G. , Munganga, B. P. , Tang, Y. K. , & Xu, P. (2021). Dynamic changes in microbial community structure in farming pond water and their effect on the intestinal microbial community profile in juvenile common carp (*Cyprinus carpio* L.). Genomics, 113(4), 2547–2560. 10.1016/j.ygeno.2021.05.024 34029696

[ece370283-bib-0023] Kang, W. , Kim, P. S. , Tak, E. J. , Sung, H. , Shin, N.‐R. , Hyun, D.‐W. , Whon, T. W. , Kim, H. S. , Lee, J.‐Y. , Yun, J.‐H. , Jung, M.‐J. , & Bae, J.‐W. (2022). Host phylogeny, habitat, and diet are main drivers of the cephalopod and mollusk gut microbiome. Animal Microbiome, 4(1), 30. 10.1186/s42523-022-00184-x 35527289 PMC9082898

[ece370283-bib-0024] Kim, P. S. , Shin, N. R. , Lee, J. B. , Kim, M. S. , Whon, T. W. , Hyun, D. W. , Yun, J. H. , Jung, M. J. , Kim, J. Y. , & Bae, J. W. (2021). Host habitat is the major determinant of the gut microbiome of fish. Microbiome, 9(1), 166. 10.1186/s40168-021-01113-x 34332628 PMC8325807

[ece370283-bib-0025] Kirchoff, N. S. , Cornwell, T. , Stein, S. , Clements, S. , & Sharpton, T. J. (2022). Gut microbial composition of Pacific salmonids differs across Oregon River basins and hatchery ancestry. Microorganisms, 10(5), 933. 10.3390/microorganisms10050933 35630377 PMC9144809

[ece370283-bib-0026] Kokou, F. , Sasson, G. , Friedman, J. , Eyal, S. , Ovadia, O. , Harpaz, S. , Cnaani, A. , & Mizrahi, I. (2019). Core gut microbial communities are maintained by beneficial interactions and strain variability in fish. Nature Microbiology, 4(12), 2456–2465. 10.1038/s41564-019-0560-0 31548685

[ece370283-bib-0027] Li, L. H. , Lv, S. , Lu, Y. , Bi, D. Q. , Guo, Y. H. , Wu, J. T. , Yue, Z. Y. , Mao, G. Y. , Guo, Z. X. , Zhang, Y. , & Tang, Y. F. (2019). Spatial structure of the microbiome in the gut of *Pomacea canaliculata* . BMC Microbiology, 19(1), 273. 10.1186/s12866-019-1661-x 31805864 PMC6896589

[ece370283-bib-0028] Li, S. X. , Qian, Z. J. , Yang, J. N. , Lin, Y. F. , Li, H. , & Chen, L. (2022). Seasonal variation in structure and function of gut microbiota in. Ecology and Evolution, 12(8), e9162. 10.1002/ece3.9162 35919391 PMC9336170

[ece370283-bib-0029] Lilli, G. , Sirot, C. , Campbell, H. , Brophy, D. , Graham, C. T. , & George, I. F. (2023). Geographic origin and host's phylogeny are predictors of the gut mucosal microbiota diversity and composition in Mediterranean scorpionfishes (*Scorpaena* spp.). Frontiers in Marine Science, 10, 1286706. 10.3389/fmars.2023.1286706

[ece370283-bib-0030] Liu, H. F. , Yang, X. M. , Yang, W. , Zheng, Z. M. , & Zhu, J. Y. (2023). Gut microbiota of freshwater gastropod (*Bellamya aeruginosa*) assist the adaptation of host to toxic cyanobacterial stress. Toxins, 15(4), 252. 10.3390/toxins15040252 37104190 PMC10141019

[ece370283-bib-0031] Liu, Y. , He, L. , Hilt, S. , Wang, R. , Zhang, H. , & Ge, G. (2020). Shallow lakes at risk: Nutrient enrichment enhances top‐down control of macrophytes by invasive herbivorous snails. Freshwater Biology, 66(3), 436–446. 10.1111/fwb.13649

[ece370283-bib-0032] Nicolai, A. , Rouland‐Lefevre, C. , Ansart, A. , Filser, J. , Lenz, R. , Pando, A. , & Charrier, M. (2015). Inter‐population differences and seasonal dynamic of the bacterial gut Community in the Endangered Land Snail Helix pomatia (Gastropoda: Helicidae). Malacologia, 59(1), 177–190. 10.4002/040.059.0101

[ece370283-bib-0033] Pawar, K. D. , Dar, M. A. , Rajput, B. P. , & Kulkarni, G. J. (2015). Enrichment and identification of cellulolytic bacteria from the gastrointestinal tract of Giant African snail, *Achatina fulica* . Applied Biochemistry and Biotechnology, 175(4), 1971–1980. 10.1007/s12010-014-1379-z 25432338

[ece370283-bib-0034] Picazo, F. , Vilmi, A. , Aalto, J. , Soininen, J. , Casamayor, E. O. , Liu, Y. Q. , Wu, Q. L. , Ren, L. J. , Zhou, J. Z. , Shen, J. , & Wang, J. J. (2020). Climate mediates continental scale patterns of stream microbial functional diversity. Microbiome, 8(1), 92. 10.1186/s40168-020-00873-2 32534595 PMC7293791

[ece370283-bib-0035] Qin, Z. , Wu, R. S. , Zhang, J. , Deng, Z. X. , Zhang, C. X. , & Guo, J. (2020). Survivorship of geographical *Pomacea canaliculata* populations in responses to cold acclimation. Ecology and Evolution, 10(8), 3715–3726. 10.1002/ece3.6162 32313630 PMC7160176

[ece370283-bib-0036] Ringø, E. , Zhou, Z. , Vecino, J. L. G. , Wadsworth, S. , Romero, J. , Krogdahl, Å. , Olsen, R. E. , Dimitroglou, A. , Foey, A. , Davies, S. , Owen, M. , Lauzon, H. L. , Martinsen, L. L. , Schryver, P. D. , Bossier, P. , Sperstad, S. , & Merrifield, D. L. (2015). Effect of dietary components on the gut microbiota of aquatic animals. A never‐ending story? Aquaculture Nutrition, 22(2), 219–282. 10.1111/anu.12346

[ece370283-bib-0037] Rudi, K. , Angell, I. L. , Pope, P. B. , Vik, J. O. , Sandve, S. R. , & Snipen, L. G. (2018). Stable Core gut microbiota across the freshwater‐to‐saltwater transition for farmed Atlantic Salmon. Applied and Environmental Microbiology, 84(2), e01974‐17. 10.1128/AEM.01974-17 29101198 PMC5752857

[ece370283-bib-0038] Sepulveda, J. , & Moeller, A. H. (2020). The effects of temperature on animal gut microbiomes. Frontiers in Microbiology, 11, 384. 10.3389/fmicb.2020.00384 32210948 PMC7076155

[ece370283-bib-0039] Servin, J. A. , Herbold, C. W. , Skophammer, R. G. , & Lake, J. A. (2007). Evidence excluding the root of the tree of life from the Actinobacteria. Molecular Biology and Evolution, 25(1), 1–4. 10.1093/molbev/msm249 18003601

[ece370283-bib-0040] Sommer, F. , Ståhlman, M. , Ilkayeva, O. , Arnemo, J. M. , Kindberg, J. , Josefsson, J. , Newgard, C. B. , Fröbert, O. , & Bäckhed, F. (2016). The gut microbiota modulates energy metabolism in the hibernating Brown bear *Ursus arctos* . Cell Reports, 14(7), 1655–1661. 10.1016/j.celrep.2016.01.026 26854221

[ece370283-bib-0041] Stock, W. , Callens, M. , Houwenhuyse, S. , Schols, R. , Goel, N. , Coone, M. , Theys, C. , Delnat, V. , Boudry, A. , Eckert, E. M. , Laspoumaderes, C. , Grossart, H. P. , De Meester, L. , Stoks, R. , Sabbe, K. , & Decaestecker, E. (2021). Human impact on symbioses between aquatic organisms and microbes. Aquatic Microbial Ecology, 87, 113–138. 10.3354/ame01973

[ece370283-bib-0042] Tamburi, N. E. , & Martín, P. R. (2009). Reaction norms of size and age at maturity of *Pomacea canaliculata* (Gastropoda: Ampullariidae) under a gradient of food deprivation. Journal of Molluscan Studies, 75(1), 19–26. 10.1093/mollus/eyn031

[ece370283-bib-0043] Tamburi, N. E. , & Martín, P. R. (2011). Effects of food availability on reproductive output, offspring quality and reproductive efficiency in the apple snail. Biological Invasions, 13(10), 2351–2360. 10.1007/s10530-011-0047-2

[ece370283-bib-0044] Thukral, A. K. (2017). A review on measurement of alpha diversity in biology. Agricultural Research Journal, 54(1), 1. 10.5958/2395-146x.2017.00001.1

[ece370283-bib-0045] Wilson, B. A. , Goertz, S. , de Menezes, A. B. , Birtles, R. J. , Fenn, J. , Lowe, A. E. , MacColl, A. D. C. , Poulin, B. , Young, S. , Bradley, J. E. , & Taylor, C. H. (2019). Geographical location influences the composition of the gut microbiota in wild house mice (*Mus musculus domesticus*) at a fine spatial scale. PLoS One, 14(9), e0222501. 10.1371/journal.pone.0222501 31557179 PMC6767902

[ece370283-bib-0046] Xavier, R. , Soares, M. C. , Silva, S. M. , Banha, F. , Gama, M. , Ribeiro, L. , Anastácio, P. , & Cardoso, S. C. (2021). Environment and host‐related factors modulate gut and carapace bacterial diversity of the invasive red swamp crayfish (*Procambarus clarkii*). Hydrobiologia, 848(17), 4045–4057. 10.1007/s10750-021-04623-9

[ece370283-bib-0047] Xie, W. J. , Yan, Y. , Hu, J. , Dong, P. S. , Hou, D. D. , Zhang, H. J. , Yao, Z. Y. , Zhu, X. Y. , & Zhang, D. M. (2021). Ecological dynamics and Co‐occurrences among prokaryotes and microeukaryotes in a diatom bloom process in Xiangshan Bay, China. Microbial Ecology, 84(3), 746–758. 10.1007/s00248-021-01899-1 34665286

[ece370283-bib-0048] Yang, Q. , Liu, S. , He, C. , Cowie, R. H. , Yu, X. , & Hayes, K. A. (2019). Invisible apple snail invasions: Importance of continued vigilance and rigorous taxonomic assessments. Pest Management Science, 75(5), 1277–1286. 10.1002/ps.5241 30324686

[ece370283-bib-0049] Yoshie, H. , & Yusa, Y. (2008). Effects of predation on the exotic freshwater snail *Pomacea canaliculata* (Caenogastropoda: Ampullariidae) by the indigenous turtle *Chinemys reevesii* (Testudines: Geoemydidae). Applied Entomology and Zoology, 43(4), 475–482. 10.1303/aez.2008.475

[ece370283-bib-0050] Yuan, M. M. , Guo, X. , Wu, L. , Zhang, Y. , Xiao, N. , Ning, D. , Shi, Z. , Zhou, X. , Wu, L. , Yang, Y. , Tiedje, J. M. , & Zhou, J. (2021). Climate warming enhances microbial network complexity and stability. Nature Climate Change, 11(4), 343–348. 10.1038/s41558-021-00989-9

[ece370283-bib-0051] Zhang, Z. Y. , Fan, Z. J. , Yi, M. M. , Liu, Z. G. , Ke, X. L. , Gao, F. Y. , Cao, J. M. , Wang, M. , Chen, G. , & Lu, M. X. (2022). Characterization of the core gut microbiota of Nile tilapia (*Oreochromis niloticus*): Indication of a putative novel *Cetobacterium* species and analysis of its potential function on nutrition. Archives of Microbiology, 204(12), 690. 10.1007/s00203-022-03301-1 36326884

[ece370283-bib-0052] Zheng, D. P. , Liwinski, T. , & Elinav, E. (2020). Interaction between microbiota and immunity in health and disease. Cell Research, 30(6), 492–506. 10.1038/s41422-020-0332-7 32433595 PMC7264227

[ece370283-bib-0053] Zhou, Z. H. , Wu, H. Y. , Li, D. H. , Zeng, W. L. , Huang, J. L. , & Wu, Z. J. (2022). Comparison of gut microbiome in the Chinese mud snail (*Cipangopaludina chinensis*) and the invasive golden apple snail (*Pomacea canaliculata*). PeerJ, 10, e13245. 10.7717/peerj.13245 35402093 PMC8992660

