# Supplementary material for: Structural and functional differences of gut microbiota in Pomacea canaliculata from different geographical locations and habitats
Source: Ecol Evol. 2024 Oct 3;14(10):e70283. doi: 10.1002/ece3.70283 (PMC11447369; doi:10.1002/ece3.70283)
Supplement: Supplementary file 1 — Data S1. [file ECE3-14-e70283-s001.docx]

**Structural and functional differences of gut microbiota in *Pomacea canaliculata* from different geographical locations and habitats**

Miao Fang^1, 2, 3^, Fandong Yu ^1,2, 3^, Lu Shu^1, 2, 3^, Hui Wei^1, 2,3^, Xidong Mu^1, 2, 3^, Xuejie Wang^1, 2, 3^, Meng Xu^*1, 2, 3^, Dangen Gu^*1, 2, 3^

^1^Pearl River Fisheries Research Institute, Chinese Academy of Fishery Sciences, Guangzhou, China

^2^Key Laboratory of Prevention and Control for Aquatic Invasive Alien Species, Ministry of Agriculture and Rural Affairs, Guangzhou, China

^3^Key Laboratory of Alien Species and Ecological Security (CAFS), Chinese Academy of Fishery Sciences, Guangzhou, China

* Corresponding author

Meng Xu, Email: [xumeng@prfri.ac.cn](mailto:xumeng@prfri.ac.cn); Dangen Gu, Email: [gudangen@163.com](mailto:gudangen@163.com)

**Table S1** ANOVA results of models for analyzing geographical locations (grl), habitats (hs) and interaction between grl and hs effects on the alpha diversity of *P. canaliculata* including Shannon index, Chao1 index, Simpson index, and relative abundance of metabolism gene, genetic information processing gene, environmental information processing gene, cellular processes gene, human diseases gene, organismal systems gene, unclassified gene, respectively.

| **Response variable** | **Fixed term** | **Df** | **Ddf** | **F** | ***P*** |
| --- | --- | --- | --- | --- | --- |
| Shannon index | grl | 4  2  8  4  2  8  4  2  4  2  8  4  2  8  4  2  8  4  2  8  4 | 208 | 24.873 | **<0.001** |
|  | hs |  | 208 | 8.132 | **<0.001** |
|  | grl : hs |  | 208 | 1.988 | **0.049** |
| Chao1 index | grl |  | 208 | 22.579 | **<0.001** |
|  | hs |  | 208 | 6.324 | **0.002** |
|  | grl : hs |  | 208 | 3.879 | **<0.001** |
| Simpson index | grl |  | 216 | 14.349 | **<0.001** |
|  | hs |  | 216 | 3.168 | 0.102 |
| Relative abundance of | grl |  | 210 | 36.482 | **<0.001** |
| metabolism gene | hs |  | 210 | 3.168 | **0.044** |
|  | grl : hs |  | 210 | 4.679 | **<0.001** |
| Relative abundance of | grl |  | 210 | 46.623 | **<0.001** |
| genetic information | hs |  | 210 | 2.072 | 0.129 |
| processing gene | grl : hs |  | 210 | 4.203 | **<0.001** |
| Relative abundance of | grl |  | 210 | 52.482 | **<0.001** |
| environmental information | hs |  | 210 | 2.701 | 0.069 |
| processing gene | grl : hs |  | 210 | 5.571 | **<0.001** |
| Relative abundance of | grl |  | 210 | 6.440 | **<0.001** |
| cellular processes gene | hs |  | 210 | 3.384 | 0.138 |
| Relative abundance of | grl : hs  grl |  | 210  210 | 2.517  13.070 | **<0.001**  **<0.001** |
| human diseases gene | hs | 2 | 210 | 6.409 | **0.002** |
|  | grl : hs | 8 | 210 | 5.250 | **<0.001** |
| Relative abundance of | grl | 4 | 208 | 43.593 | **<0.001** |
| organismal systems gene | hs | 2 | 208 | 2.107 | 0.124 |
|  | grl : hs | 8 | 208 | 7.108 | **<0.001** |
| Relative abundance of | grl | 4 | 210 | 44.502 | **<0.001** |
| unclassified gene | hs | 2 | 210 | 2.348 | 0.098 |
|  | grl : hs | 8 | 210 | 5.195 | **<0.001** |

Df = numerator degrees of freedom of the fixed term; Ddf = denominator degrees of freedom of the fixed term. Significance (*P* < 0.05) was highlighted in bold fonts.

**
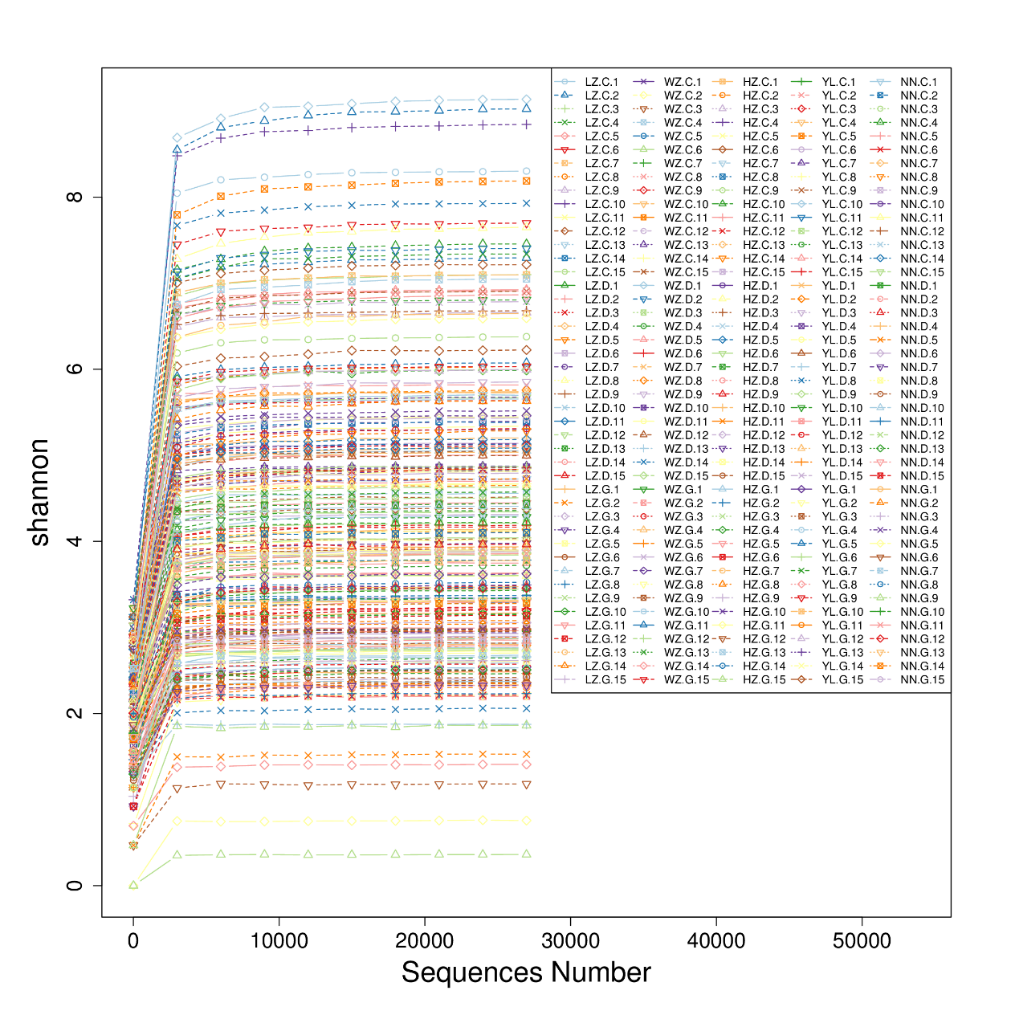
**

**Figure S1** The rarefaction curves of all samples of *P. canaliculata*

**Table S2** Sampling information of different geographical locations and habitats

| Sites | Habitats | Number of individuals | Body Weight (g)  (mean±se) | Shell height (cm)  (mean±se) | Sampling  date | Sampling location |
| --- | --- | --- | --- | --- | --- | --- |
| HZ | ditch | 15 | 18.35 ± 2.26 | 4.75 ± 0.16 | August  2022 | 111°67′E, 24°35′N |
| HZ | paddy | 15 | 9.95 ± 0.68 | 3.81 ± 0.10 |  |  |
| HZ | pond | 15 | 16.81 ± 1.49 | 4.63 ± 0.13 |  |  |
| LZ | ditch | 15 | 11.87 ± 0.78 | 4.09 ± 0.07 | September  2022 | 109°31′E, 24°37′N |
| LZ | paddy | 15 | 9.81 ± 0.46 | 3.88 ± 0.05 |  |  |
| LZ | pond | 15 | 14.68 ± 1.38 | 4.64 ± 0.14 |  |  |
| NN | ditch | 15 | 13.20 ± 1.04 | 4.09 ± 0.11 | September  2022 | 107°77′E, 23°09′N |
| NN | paddy | 15 | 12.79 ± 0.74 | 4.10 ± 0.08 |  |  |
| NN | pond | 15 | 17.26 ± 1.26 | 4.48 ± 0.10 |  |  |
| WZ | ditch | 15 | 13.58 ± 0.90 | 4.10 ± 0.09 | August  2022 | 110°30′E, 23°54′N |
| WZ | paddy | 15 | 10.63 ± 0.84 | 3.88 ± 0.10 |  |  |
| WZ | pond | 15 | 27.73 ± 2.68 | 5.32 ± 0.15 |  |  |
| YL | ditch | 15 | 8.80 ± 0.63 | 3.59 ± 0.07 | September  2022 | 109°99′E, 22°32′N |
| YL | paddy | 15 | 8.67 ± 0.41 | 3.60 ± 0.06 |  |  |
| YL | pond | 15 | 21.36 ± 1.39 | 5.01 ± 0.12 |  |  |

**
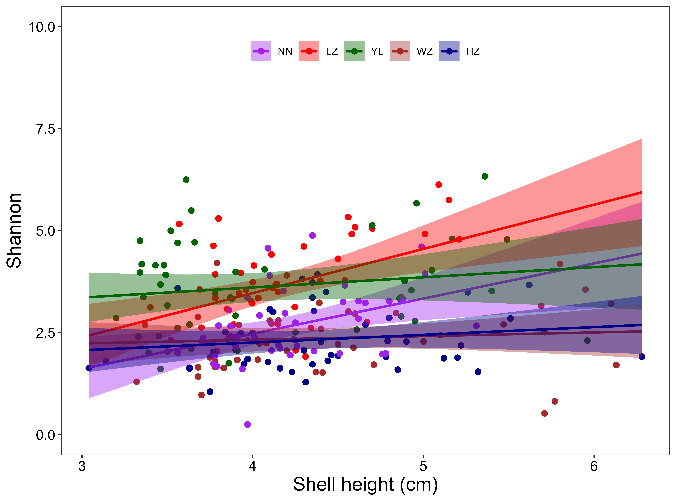

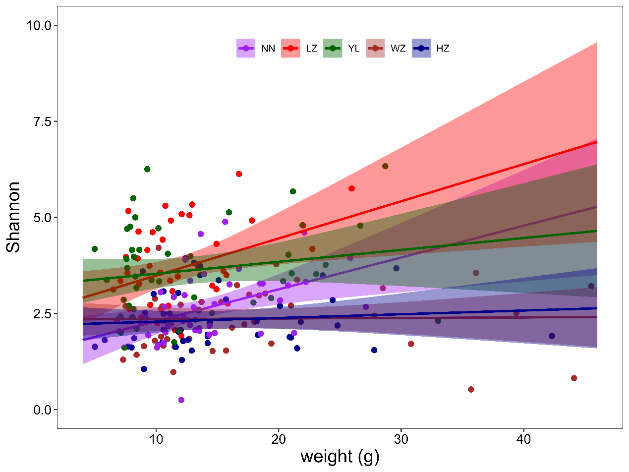
**

**Figure S2** The relationship between *P. canaliculata* body weight and shell height, Shannon index

**
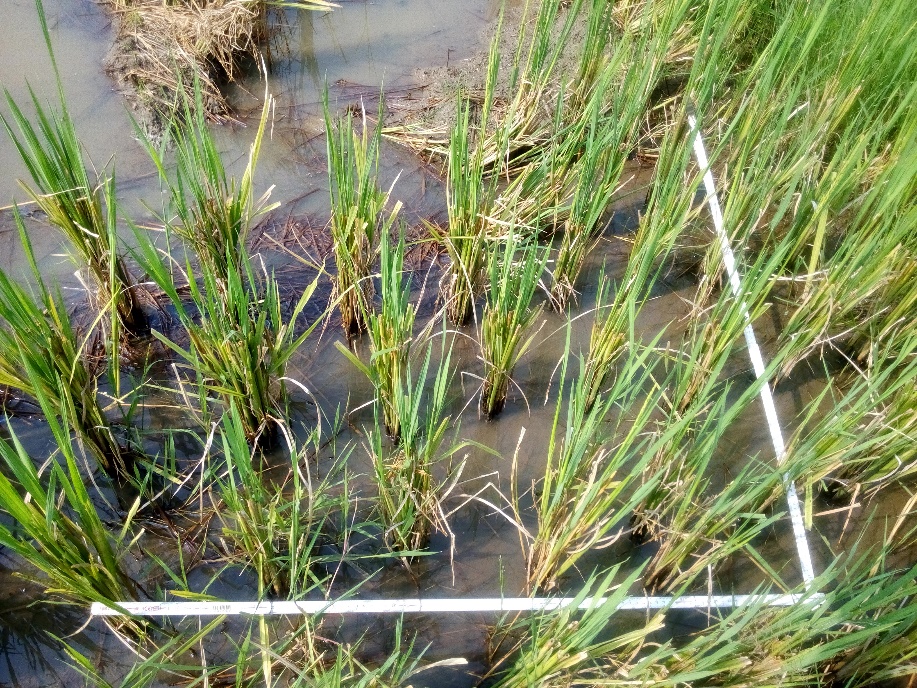
**

**Figure S3** Sampling quadrats (1m^2^) in the field.
